# Monitoring of (Leukemia-Specific) Immune Cells in Stages, Treatment Groups and in the Course of Disease and Therapy Contributes to Qualify Antileukemic Potential and Survival in Patients with AML

**DOI:** 10.3390/ijms262110336

**Published:** 2025-10-23

**Authors:** Julian Stein, Philipp Anand, Joudi Abdulmajid, Anne Hartz, Marianne Unterfrauner, Xiaojia Feng, Nicolas Schmieder, Linus Kruk, Peter Bojko, Joerg Schmohl, Christoph Schmid, Giuliano Filippini Velázquez, Helga M. Schmetzer

**Affiliations:** 1 Department of Medicine III, University Hospital of Munich, Immunomodulation, Marchioninistr. 15, 81377 Munich, Germany; stein.julian@web.de (J.S.); philippanand@gmail.com (P.A.); joudiabdulmajid9589@hotmail.com (J.A.); annsohart@gmail.com (A.H.); marianne.unterfrauner@yahoo.de (M.U.); xiaojia.feng@med.uni-muenchen.de (X.F.); nicolas.schmieder@icloud.com (N.S.); linus.kruk@med.uni-muenchen.de (L.K.); 2 Bavarian Cancer Research Center (BZKF) Comprehensive Cancer Center, 91054 Erlangen, Germany; christoph.schmid@uk-augsburg.de (C.S.); giuliano.filippinivelazquez@uk-augsburg.de (G.F.V.); 3 Faculty of Biology, Department of RNA Biology and Molecular Physiology, Bielefeld University, 33615 Bielefeld, Germany; 4 Department of Hematology and Oncology, Rotkreuzklinikum Munich, 80634 Munich, Germany; peter.bojko@swmbrk.de; 5 Department of Hematology and Oncology, Diakonieklinikum Stuttgart, 70176 Stuttgart, Germany; joerg.schmohl@diak-stuttgart.de; 6 Department of Hematology and Oncology, University Hospital of Augsburg, 86156 Augsburg, Germany

**Keywords:** AML, leukemia specific cells, immunomodulation, immune-monitoring, leukemia therapy, prognosis, response to induction therapy, survival

## Abstract

Various AML treatment regimens might trigger different immunological mechanisms against leukemic cells. The role of different immune cell subsets in the mediation of antileukemic processes is not clear. In this study, we longitudinally assessed (leukemia specific) immune subtype compositions in 17 AML patients before stem cell transplantation (SCT) at different timepoints in the course and in different stages of the disease using flow cytometry. Further we correlated immune cell compositions with patients’ response to induction therapy and the median survival (3.8 months in our cohort) of the patients. Finally, we compared immune cell profiles from patients before and after SCT. (1) Patients in CR (compared to dgn and PD) were characterized by higher frequencies of leukemia-derived DC (DCleu), (leukemia-specific—IFNg or TNFα producing or CD107a degranulating) anti-tumor relevant T cells (Tgd, Tβ7), central/effector memory cells (Tcm, Tem), alongside with lower frequencies of (leukemia-specific) regulatory T cells. (2) Patients with higher frequencies of (leukemia-specific) antitumor relevant T cells, (leukemia-specific) memory T cells and NK cells demonstrated a prolonged median survival time and/or responded better to induction (RTI) treatment (3) Comparing patients before and after SCT, only minimal differences were observed. However, patients in CR_preSCT_ exhibited higher frequencies of DC, Tcm, Tβ7 and leukemia-specific iNKT cells compared to patients in CR_postSCT_. (1) Immune monitoring qualifies to quantify (leukemia-specific) immune cells in different stages and under different treatment strategies in the course of AML. (2) Higher frequencies of activating and antitumor relevant leukemia-specific immune cell subtypes found after ‘costimulatory’ (especially KitM induced) treatment’ and in CR. (3) In particular, DC/DCleu, (leukemia-specific) antitumor-relevant T (memory) and NK cells seem to dominate in CR and positively influence RTI and survival. (4) Monitoring of (leukemia-specific) immune cell subtypes contribute to quantify individual AML patients’ antileukemic potential in different stages and treatment groups and also could be used to predict patients’ survival.

## 1. Introduction

### 1.1. Acute Myeloid Leukemia (AML)

Acute myeloid leukemia is a hematologic malignancy characterized by maturation arrest in the myeloid lineage, resulting in consecutive clonal expansion and accumulation of myeloid precursor cells (e.g., CD34^+^ or CD117^+^ blasts). The risk classification is primary based on the patients’ age, cytogenetic aberrations and is commonly assessed using the European Leukemia Net (ELN) score [1,2]. According to epidemiological data from the United States, the median age at diagnosis is 69 years and has a 5-year survival rate of 31.9% between 2014 and 2020 [3].

Chemotherapeutic induction with cytarabine and anthracycline leads to a disease response in 50–80% of patients, depending on their allocation to favorable or intermediate ELN risk groups. In contrast, patients classified as adverse ELN risk are characterized by lower complete remission (CR) rates of approximately 40%. Induction treatment is followed by a consolidation phase and at least two cycles of high dose cytarabine [4,5]. Targeted treatment with midostaurin addresses FMS-like tyrosine kinase 3 gene (FLT3) mutations (found in 35% of patients) and leads to increased CR rates in combination with conventional chemotherapy [6]. Elderly and insufficiently resilient patients are treated with hypomethylating agents (HMA) such as decitabine or azacitidine. Combinations of HMA with BCL-2 inhibitor venetoclax increases patients’ response and remission rates due to more efficient myelosuppression [7,8]. Gemtuzumab ozogamicin (GO), an antibody-drug conjugate, targets CD33 expressing myeloid and blast cells, thereby enhancing apoptosis and reducing the risk of relapse in AML [9,10]. DC based immunotherapy represents another therapeutic approach and involves the ex-vivo generation of monocyte-derived DCs loaded with leukemia-associated antigens or of leukemia-derived DCs (DCleu) that have to be adoptively administered to the patient [11,12]. Alternatively, DCleu can be generated (ex and in vivo) from blasts using KitM (GM-CSF and prostaglandin E1) and have been shown to induce (leukemia-specific) immune cells in vivo in therapy refractory patients after treatment in an off-label use [13,14].

Due to the high risk of relapse, patients receive maintenance therapy to stabilize their CR, e.g., low-dose chemotherapy, targeted therapy targeting detectable mutations or allogeneic stem cell transplantation (SCT) as preferred curative treatment, especially for high-risk patients [15].

### 1.2. Cells of the Innate and Adaptive Immune System

#### 1.2.1. Innate Immune System

The innate immune system, with key components including CIK, NK, and iNKT cells, plays an important role in the early detection and elimination of pathogens and malignant tumor, including leukemic cells [16,17,18]. Natural killer cells (NK, CD3^−^CD56^+^) and cytokine-induced killer cells (CIK, CD3^+^CD56^+^) show cytotoxic activity, inhibiting the migration and proliferation of tumor cells [18,19]. It has also been shown that the absolute number of CIK cells is increased in blood of AML patients at diagnosis and returns to normal levels in CR [20]. Invariant natural killer T cells (iNKT, 6B11^+^) combine T and NK cell properties, activate B and mature dendritic cells and can release both pro-inflammatory (Th_1_) and anti-inflammatory (Th_2_) cytokines [21,22].

Dendritic cells (DC, CD80^+^ or CD206^+^) serve as antigen-presenting cells (APC), connect innate/adaptive immunity and play an important role in the development of immunological memory and tolerance within lymphoid organs [23,24]. Leukemia-derived DC (DCleu, DC^+^Bla^+^) present leukemic antigens to immune cells in a costimulatory manner [25]. They can be generated in vitro or can be induced in vivo from blasts, specifically activate leukemia specific cells—leading to improved immune responses against myeloid leukemia [25]. The composition of innate immune cells and cell subsets is provided in Table 1.

#### 1.2.2. Adaptive Immune System

Non-naive T cells (Tnn, CD3^+^CD45RO^+^) and their subgroups (central memory cells (Tcm, CD3^+^CD45RO^+^CD197^+^) and effector memory cells (Tem, CD3^+^CD45RO^+^CD197^−^)) mediate effector functions after first and after further antigen contact as memory cells [25].

γδ-expressing T cells (Tgd, CD3^+^TCRγδ^+^) as well as integrin expressing β7 T cells (Tβ7, CD3^+^Intβ7^+^) play an important role in the activation and subsequent proliferation of (antitumor directed) T cells by releasing cytokines [31,36].

CD4 positive T cells (T4^+^, CD3^+^CD4^+^) assist in the production of antibodies by B cells, regulate macrophages and are important mediators of immunological memory (Th1 and Th2 immune response) [38]. Regulatory T cells (Treg, CD3^+^CD4^+^CD25^++^CD127^(+)^) control immune responses, maintain immunological self-tolerance [32] and are known to down regulate antitumor response [39].

T cell receptor (TCR) binding to the MHC of the APC needs a costimulatory signal, provided by T cells with 4-1BB (T137, CD3^+^CD137^+^) or CD40L (T154, CD3^+^CD154^+^), leading to the differentiation and proliferation of T137, while T154 positively influences both the humoral and cell-based immune response [35,40].

Downregulation of T cell immune responses are mediated by upregulated CTLA4 (T152, CD3^+^CD152^+^) and downregulated CD28 after contact with (CD80^+^) DC [33,41]. The composition of adaptive immune cells and cell subsets is provided in Table 1.

### 1.3. Leukemia-Specific Cells

Leukemia-specific cells are specifically triggered immune cells that produce interferon gamma (IFNg), tumor necrosis factor α (TNFα) or initiate cells’ degranulation in the presence of leukemia-associated antigens (LAA) like WT1 or PRAME [25,42].

Frequencies of TNFα- and IFNg-producing innate (NK_IFNg_, CIK_IFNg_) or adaptive (T_IFNg_, T4^+^_IFNg_, T4^−^_IFNg_, Tnn_IFNg_, Tem_IFNg_, Tcm_IFNg_, Tgd_TNFα_, Tβ7_IFNg_) immune cells (after LAA stimulation) can be monitored using the intracellular cytokine assay (INCYT) [25,42].

Degranulation of innate (NK_107a_, CIK_107a_, iNKT_107a_) or adaptive (T_107a_, Tnn_107a_, Tem_107a_, Tcm_107a_, Tgd_107a_, Tβ7_107a_, Treg_107a_) lymphocytes (after LAA stimulation) can be detected by lysosome-associated membrane protein-1 (LAMP-1, CD107a) [25]. The composition of leukemia-specific immune cells and cell subsets is provided in Table 1.

### 1.4. Aims of This Study

We analyzed frequencies of (leukemia-specific) innate and adaptive immune cells in AML patients without SCT in different stages, in different treatment groups and over the course of the disease and correlated these frequencies with disease characteristics, response to induction therapy and the median survival time of the patients to contribute to define risk groups for response to treatment, to predict relapses or to detect treatment associated effects. Furthermore, we compared immune cell profiles of patients before and after SCT to detect SCT-related differences in the composition of immune cells of AML patients. Finally, we want to contribute to an improved immune monitoring and prognostic classification.

## 2. Results

### 2.1. Composition of Blood Cells in AML Patients in Different Stages or Treatment Groups

We compared the composition of blood cells in different stages and treatment groups of AML patients.

#### 2.1.1. Composition of Blasts and DC Subtypes

**Figure 1:**
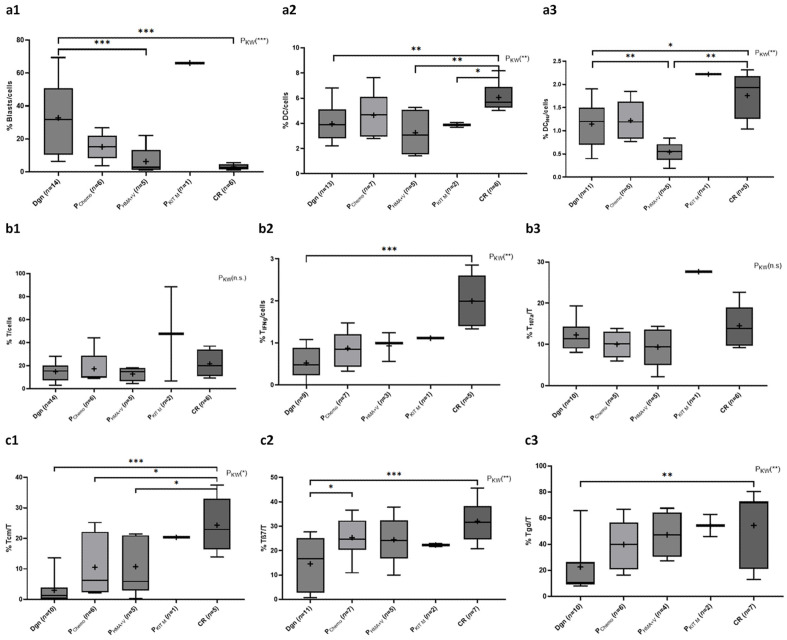

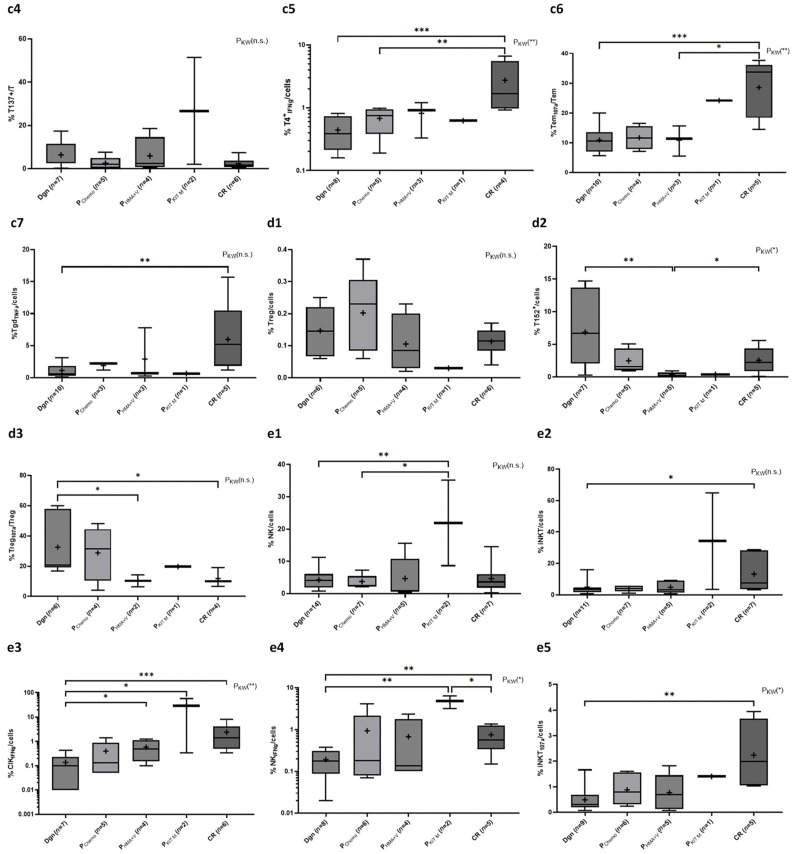
Given are median (—) and mean frequencies (+) ± standard deviations of myeloid (**a**,**a1**–**a3**) and immune cells ((**b**,**b1**–**b3**): T cells, (**c**,**c1**–**c7**): effector and memory cells, (**d**,**d1**–**d3**): regulatory cells, (**e**,**e1**–**e5**): innate immune cells) in the groups (diagnosis (dgn), persisting disease (PD) under treatment or in CR). Abbreviations for cell subtypes are given in Table 1, explanations for treatment groups are given in Table 2. Statistical analyses were conducted using the non-parametric Kruskal-Wallis (P_KW_) test to compare all groups and the Mann-Whitney U-test to compare two groups. Differences were considered as highly significantly different with *p*-values ≤ 0.005 (***), as significantly different with *p*-values ≤ 0.05 (**) and as borderline significantly different with *p*-values ≤ 0.1 (*). Non-significant values within P_KW_ with *p*-values > 0.1 were considered as n.s. Results in figures (**c5**,**e3**,**e4**) are displayed logarithmically (log10) for improved presentation.

Results of Kruskal-Wallis test showed highly significant differences in the groups compared. We found significantly higher frequencies of blasts at dgn compared to persistent disease (PD) and to CR (e.g., %blasts/cells: dgn 32.8 ± 21.6 vs. CR 1.9 ± 1.6, *p* < 0.0001) Figure 1a). Frequencies of DC/cells and DCleu/cells were (borderline) significantly lower in patients at dgn compared to CR (e.g., %DC/cells: dgn 4.0 ± 1.3 vs. CR 6.1 ± 1.1, *p* = 0.0088) (Figure 1a). Patients with PD treated with KitM presented with higher frequencies of blasts (Figure 1a), but also higher frequencies of DCleu/cells (Figure 1a).

**Table 2:** Composition of treatment and stage groups studied in the course of the disease. Treatment and stage groups before SCT. Treatment and/stage groups after SCT. The cohort after SCT is used exclusively for the comparative analysis in Section 3.6.

Name of Treatment Groups	Abbreviation	Treatment at Timepoint of Analysis
First diagnosis	Dgn	- no treatment (*n* = 14)
Persisting disease (PD) under chemotherapy (before allogeneic stem cell transplantation)	P_Chemo_	- cytarabine and daunorubicine (*n* = 7) - or sequential high-dose cytosine arabinoside and mitoxantrone (S-HAM) (*n* = 1) - or hydroxyurea (*n* = 1) - or midostaurine (*n* = 1)
Persisting disease (PD) under hypomethylating agents and venetoclax (before allogeneic stem cell transplantation)	P_HMA+V_	- azacitidine or decitabine (*n* = 5) with venetoclax (*n* = 4)
Persisting disease (PD) under KitM	P_KitM_	- KitM (*n* = 2) with hydroxyurea (*n* = 1)
Complete remission (before allogeneic stem cell transplantation)	CR	- no treatment (*n* = 5) - KitM (*n* = 1) - Ivosidenib (*n* = 1)
Relapse after allogeneic stem cell transplantation	Rel_postSCT_	- no treatment (*n* = 14)
Relapse under hypomethylating agents and venetoclax after allogeneic stem cell transplantation	Rel_HMA+V postSCT_	- azacitidine or decitabine (*n* = 10) with venetoclax (*n* = 9)
Complete remission after allogeneic stem cell transplantation	CR_postSCT_	- no treatment (*n* = 12)

**Legend:** *n*: number of patients who have received the therapy in the course of disease and treatment. Abbreviations are supplemented with the suffix “preSCT” in Section 3.6 and corresponding figures for more precise differentiation.

#### 2.1.2. Composition of (Leukemia-Specific) Adaptive Cells of the Immune System

Frequencies of CD3^+^ T-cells in different stages of the diseases (dgn, PD, CR) were not significantly different (Figure 1b). However, (highly significantly) lower frequencies of leukemia-specific T_IFNg_/cells were found at dgn and PD compared to CR (e.g., %T_IFNg_/cells: dgn 0.6 ± 0.3 vs. CR 2.0 ± 0.5, *p* = 0.0016) (Figure 1b). Frequencies of T107a/T were lower at dgn and during PD compared to CR and high in the patient with PD during KitM treatment (Figure 1b).

We found highly significantly lower frequencies of Tcm/T (Figure 1c) at dgn compared to CR (%Tcm/T: dgn 3.0 ± 4.1 vs. CR 24.3 ± 8.1, *p* = 0.0007). Frequencies of Tcm/T in PD were comparable and higher compared to dgn but borderline significantly lower compared to CR. Within Tem_107a_/Tem, we observed highly significantly lower frequencies at dgn compared to CR (Figure 1c). Frequencies of antitumor-directed T cell subsets Tβ7/T and Tgd/T were (highly) significantly lower at dgn compared to CR (e.g., %Tβ7/T: dgn 16.4 ± 11.3 vs. CR 32.0 ± 7.8, *p* = 0.0098) (Figure 1c). Related to leukemia-specific, antitumor-directed T cell subsets (Tβ7_107a_/Tβ7 and Tgd_TNFα_/Tgd), we found (borderline) significantly lower frequencies at dgn compared to CR (e.g., %Tgd_TNFα_/Tgd: dgn 36.2 ± 20.2 vs. CR 62.4 ± 20.8, *p* = 0.0553). Moreover, we found significantly higher frequencies of Tgd_TNFα_/cells (Figure 1c) in CR compared to dgn. Frequencies of T4^+^ lymphocytes showed no significant differences between the different treatment groups, while we found (highly) significantly lower frequencies of T4^+^_IFNg_/cells at dgn and in PD compared to CR (e.g., %T4^+^_IFNg_/cells: dgn 0.4 ± 0.3 vs. CR 2.7 ± 2.3, *p* = 0.0040) (Figure 1c). Frequencies of T137/T (Figure 1c) or T152/T showed no significantly higher frequencies of immune cells at dgn or within the different treatment groups compared to CR.

Frequencies of Treg/cells were (not significantly) higher at dgn and P_Chemo_ and lower in CR and P_HMA+V_ and P_KitM_ (Figure 1d). Frequencies of Treg_107a_/Treg were (borderline significantly) lower in CR and in PD in patients with costimulatory treatment compared to dgn and P_Chemo_ (Figure 1d). We found (borderline) significantly lower frequencies of T152^+^/cells in PD compared to dgn and CR (e.g., %T152^+^/cells: dgn 6.8 ± 4.2 vs. P_HMA+V_ 0.3 ± 0.3, *p* = 0.0101) (Figure 1d).

Frequencies of NK/cells showed no significant differences within dgn, P_Chemo_, P_HMA+V_ and CR. We found significantly lower frequencies in patients at dgn compared to P_KitM_ (%NK/cells: dgn 4.2 ± 2.6 vs. P_KitM_ 21.9 ± 13.3, *p* = 0.0333) (Figure 1e). We found (highly) significantly lower frequencies of IFNg-producing NK_IFNg_/cells (Figure 1e) and CIK_IFNg_/cells (Figure 1e) at dgn compared to CR (e.g., %CIK_IFNg_/cells: dgn 0.1 ± 0.1 vs. CR 2.4 ± 2.3, *p* = 0.0023). Further, we found (borderline) significantly more NK_IFNg_/cells and CIK_IFNg_/cells in P_KitM_ compared to dgn and CR. Frequencies of iNKT/cells (Figure 1e) were comparable at dgn, P_Chemo_ and P_HMA+V_. Compared to dgn, we found borderline significantly higher frequencies of iNKT/cells in CR. The frequencies of degranulating iNKT_107a_/cells were significantly lower at dgn compared to CR (%iNKT_107a_/cells: dgn 0.5 ± 0.5 vs. CR 2.2 ± 1.2, *p* = 0.0112) (Figure 1e).

Abbreviations for cell subtypes are given in Table 1, explanations for treatment groups are given in Table 2.

### 2.2. Composition of Blood Cells in Individual Patients’ Courses of the Disease and Treatment

**Figure 2:**
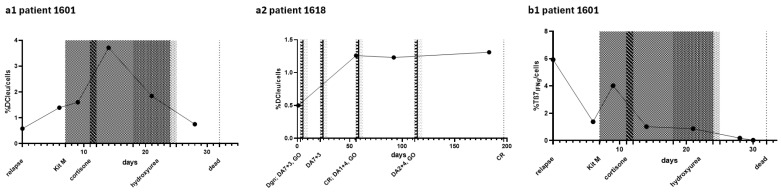

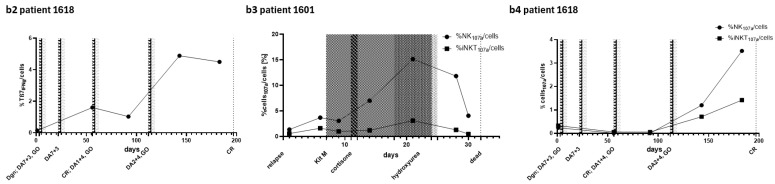
Given are frequencies of leukemia-derived dendritic cells (DCleu, (**a**,**a1**,**a2**)) and leukemia-specific innate/adaptive immune cells (**b**,**b1**–**b4**) in the course of the disease and under treatment (graphically indicated by the shadows above the therapies described within the figures) in patients 1601 and 1618. Abbreviations for cell subtypes are given in Table 1. Legend: DA7 + 3: cytarabine for 7d, daunorubicine for 3d; DA1 + 4: cytarabine for 4d, daunorubicine for 1d; DA2 + 4: cytarabine for 4d, daunorubicine for 2d; GO: gemtuzumab-ozogamicin; KitM: GM-CSF and prostaglandin E1; CR: complete remission.

We quantified frequencies of selected cell subtypes under various treatments of individual patients (Figure 2). Frequencies of DCleu/cells in patient 1601 showed an increase under treatment with KitM after relapse of AML (Figure 2a). A maximum of 3.71% of DCleu/cells was reached after seven days of treatment. A cortisone therapy for two days as well as termination of KitM treatment according to the patient’s decision resulted in a drop in frequencies of DCleu/cells to 0.75% and the patient’s death four days later. Patient 1618 started with comparable frequencies of DCleu/cells at dgn (0.50%) increasing to 1.26% in CR after 50d and two chemotherapies with cytarabine, anthracycline and one application of GO (Figure 2a). Frequencies of DCleu remained constant for over 100d in CR.

In patient 1601, a decrease in frequencies of Tβ7_IFNg_/cells from 5.9% to 1.4% was observed within one week after relapse. Under treatment with KitM, frequences increased to 4.0% and dropped again under cortisone, hydroxyurea (HU) treatment and progression (Figure 2b). Frequencies of NK_107a_/cells and iNKT_107a_/cells increased under KitM and HU treatment and decreased again shortly before the patient died (Figure 2b). Compared to 1601, patient 1618 showed a distinct increase in frequencies of Tβ7_IFNg_/cells from 0.15% at dgn to 4.7% in the course of four cycles of chemotherapy and in CR (Figure 2b). Frequencies of NK_107a_/cells and iNKT_107a_/cells however increased after the fourth cycle of chemotherapy (Figure 2b). Frequencies of NK_107a_/cells increased to 1.4% and frequencies of iNKT_107a_/cells increased to 3.52%.

### 2.3. Composition of Immune Cells in Patients at First Dgn with vs. Without Response to Induction Therapy (RTI)

We compared the composition of hematopoetic/immune cells in patients at first dgn who had vs. had not responded to induction therapy.

#### 2.3.1. Composition of Blasts and DC Subtypes

**Figure 3:**
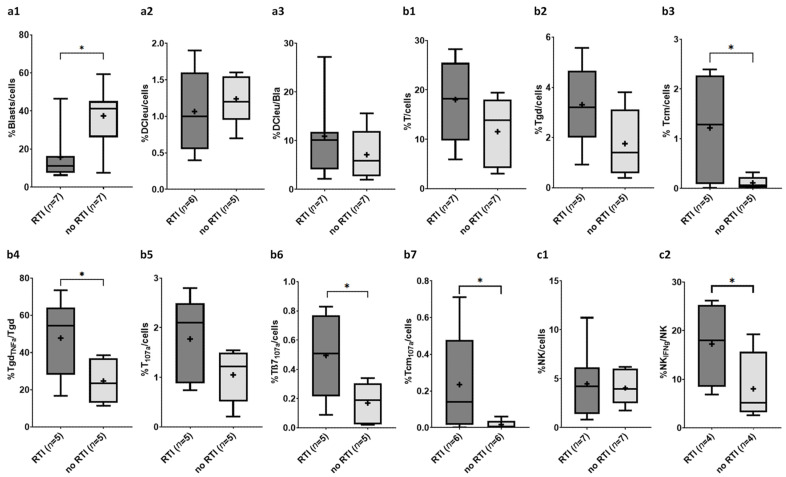
Given are median (—) and mean frequencies (+) ± standard deviations of myeloid cells (**a**,**a1**–**a3**) and (leukemia-specific) immune cells ((**b**,**b1**–**b7**) effector and memory immune cells, (**c**,**c1**,**c2**) innate immune cells) in responders to induction therapy (RTI) vs. no responders (without RTI). Statistical analyses were conducted using the non-parametric Mann-Whitney U-test to compare two groups. Differences were considered as borderline significantly different with *p*-values ≤ 0.1 (*). Abbreviations for cell subtypes are given in Table 1.

Frequencies of blasts in patients with response (RTI) vs. without response to induction therapy (without RTI) were borderline significantly lower (%blasts/cells: RTI 15.8 ± 13.9 vs. without RTI 37.5 ± 16.6, *p* = 0.0973) (Figure 3a). Frequencies of DCleu/cells were comparable in the groups with and without RTI (%DCleu/cells: RTI 1.1 ± 0.6 vs. without RTI 1.2 ± 0.4, *p* = n.s.) (Figure 3a), however frequencies of DCleu/Bla were higher in patients with RTI vs. without RTI (%DCleu/Bla: RTI 10.9 ± 7.1 vs. without RTI 7.1 ± 5.2, *p* = n.s.) (Figure 3a).

#### 2.3.2. Composition of (Leukemia-Specific) Adaptive Cells of the Immune System

We found (non-significantly) higher frequencies of T/cells, T_IFNg_/cells and T_107a_/cells in patients with RTI vs. without RTI (e.g., %T/cells: RTI 18.0 ± 8.1 vs. without RTI 11.5 ± 6.6, *p* = n.s.; T_107a_/cells: RTI 1.8 ± 0.9 vs. without RTI 1.1 ± 0.5, *p* = n.s.) (Figure 3b). Frequencies of antitumor-directed immune cells were higher in patients with RTI compared to patients without RTI (e.g., %Tgd/cells: RTI 3.3 ± 1.7 vs. without RTI 1.8 ± 1.4, *p* = n.s.) (Figure 3b). Further frequencies of leukemia-specific, antitumor-directed immune cells [Tgd_TNFα_/Tgd (Figure 3b), Tβ7_IFNg_/cells and Tβ7_107a/_cells (Figure 3b)] were borderline significantly higher in patients with RTI vs. without RTI (e.g., %Tgd_TNFα_/Tgd: RTI 47.7 ± 21.1 vs. without RTI 24.7 ± 12.1, *p* = 0.0556). Frequencies of Tcm/cells (Figure 3b), Tnn_107a_/cells and Tcm_107a_/cells (Figure 3b) in patients with RTI were borderline significantly increased compared to patients without RTI (e.g., %Tcm/cells: RTI 1.2 ± 1.1 vs. without RTI 0.1 ± 0.1, *p* = 0.0736), while frequencies of Tnn/cells were comparable.

We found no significant differences in frequencies of Treg/cells or Treg/T in patients with vs. without RTI (e.g., %Treg/cells: RTI 0.1 ± 0.1 vs. without RTI 0.1 ± 0.1, *p* = n.s.). Leukemia-specific Treg were comparable in patients with vs. without RTI as well (e.g., %Treg_107a_/Treg: RTI 23.6 ± 22.1 vs. without RTI 19.3 ± 26.6, *p* = n.s.).

Frequencies of NK/cells (Figure 3c) and CIK/cells were comparable in patients with RTI vs. without RTI (e.g., %NK/cells: RTI 4.5 ± 3.6 vs. without RTI 4.1 ± 1.7, *p* = n.s.). Frequencies of leukemia-specific subtypes [e.g., NK_IFNg_/cells, NK_IFNg_/NK (Figure 3c), CIK_107a_/cells] were (borderline significantly) higher in patients with RTI vs. without RTI (e.g., %NK_IFNg_/NK: RTI 17.3 ± 8.7 vs. without RTI 8.1 ± 7.5, *p* = 0.0857).

### 2.4. Prognostic Relevance of the Composition of Blood Cells in the Course of Treatment for Survival

We compared the compositions of blood cells in patients with longer or shorter median survival of 3.8 months in our cohort.

#### 2.4.1. Composition of Blasts and DC Subtypes

**Figure 4:**
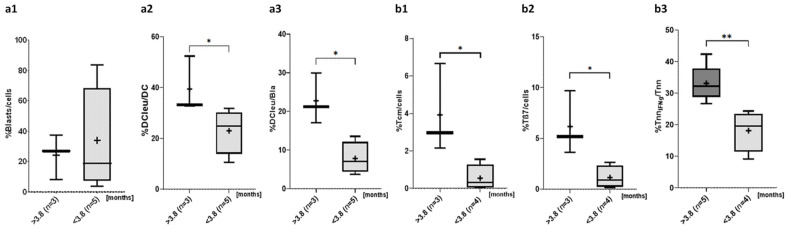
Given are median (—) and mean frequencies (+) ± standard deviations of myeloid cells (**a**,**a1**–**a3**) and (leukemia-specific) immune cells (**b**,**b1**–**b3**) in patients with a longer (>3.8 months) or shorter (<3.8 months) median survival. Statistical analyses were conducted using the non-parametric Mann-Whitney U-test to compare two groups. Differences were considered as significantly different with *p*-values ≤ 0.05 (**) and as borderline significantly different with *p*-values ≤ 0.1 (*). Abbreviations for cell subtypes are given in Table 1.

Frequencies of blasts were lower in the cohort with longer vs. shorter survival (%blasts/cells: survival > 3.8months 24.1 ± 14.9 vs. survival < 3.8 months 34.0 ± 33.7, *p* = n.s.; Figure 4a) and frequencies of DCleu/DC and DCleu/Bla were higher in the cohort with longer survival (e.g., %DCleu/DC: survival > 3.8 months 39.5 ± 11.2 vs. survival < 3.8 months 23.0 ± 9.0, *p* = 0.0571) (Figure 4a), while DC/cells were distributed homogenously in the survival groups compared (%DC/cells: survival > 3.8 months 4.4 ± 2.3 vs. survival < 3.8 months 4.0 ± 0.9, *p* = n.s.).

#### 2.4.2. Composition of (Leukemia-Specific) Activating/Antitumor-Directed T Cells of the Immune System

Although frequencies of T/cells were lower in patients with longer vs. shorter survival (%T/cells: survival > 3.8 months 16.1 ± 7.1 vs. survival < 3.8 months 32.6 ± 32.3, *p* = n.s.), frequencies of Tnn/cells, Tcm/cells (Figure 4b), Tcm/T and leukemia-specific Tnn_IFNg_/Tnn (Figure 4b), as well as of antitumor-directed immune cells Tβ7/cells (Figure 4b), Tβ7/T, Tgd/cells and Tgd/T were ((borderline) significantly) higher in patients with longer survival (e.g., %Tnn_IFNg_/Tnn: survival > 3.8 months 33.1 ± 5.7 vs. survival < 3.8 months 18.1 ± 6.5, *p* = 0.0149; Tβ7/cells: survival > 3.8 months 6.2 ± 3.2 vs. survival < 3.8 months 1.2 ± 1.1, *p* = 0.0571).

### 2.5. Comparison of Blood Cells in AML Patients in Different Stages or Treatment Groups Before vs. After SCT

We compared composition of myeloid and immune cells in patients’ blood before vs. after SCT.

#### 2.5.1. Composition of Blasts and DC Subtypes

**Figure 5:**
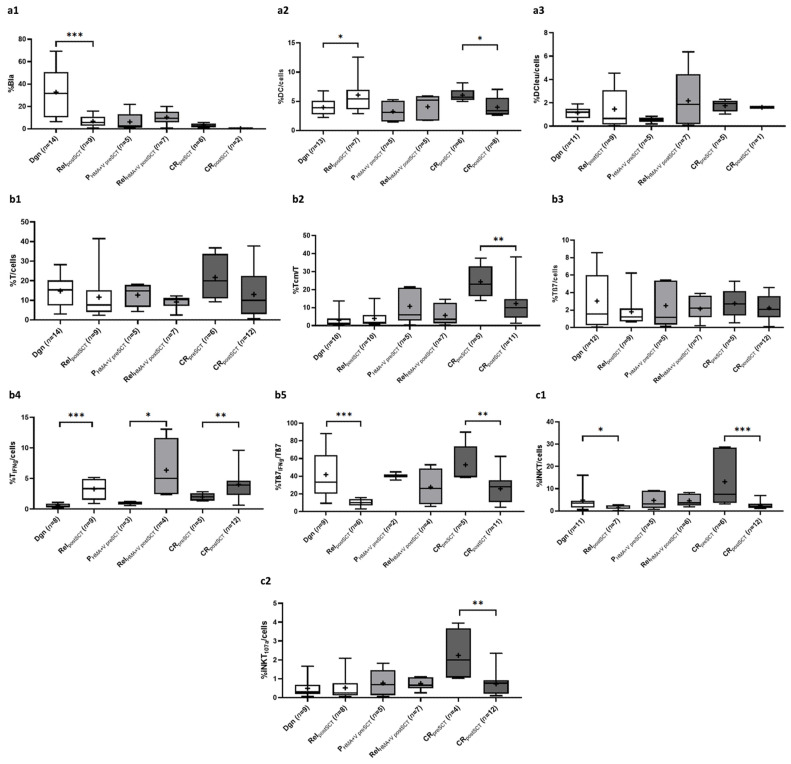
Given are median (—) and mean frequencies (+) ± standard deviations of myeloid cells (**a**,**a1**–**a3**) and (leukemia-specific) immune cells ((**b**,**b1**–**b5**) effector and memory cells, (**c**,**c1**,**c2**) innate immune cells) of patients in the course of the disease and in different stages of treatment groups before and after allogeneic stem cell transplantation. Statistical analyses were conducted using the non-parametric Mann-Whitney U-test to compare two groups. Differences were considered as highly significantly different with *p*-values ≤ 0.005 (***), as significantly different with *p*-values ≤ 0.05 (**) and as borderline significantly different with *p*-values ≤ 0.1 (*). Abbreviations for PHMA + V and CR groups before SCT were supplemented with the suffix “preSCT” for more precise differentiation. Abbreviations for cell subtypes are given in Table 1, explanations for treatment groups are given in Table 2.

Frequencies of blasts were highly significantly higher in patients at dgn compared to patients with relapse after SCT (rel_postSCT_): %Bla/cells: dgn 32.8 ± 22.4 vs. rel_postSCT_ 6.8 ± 5.2, *p* = 0.0006) (Figure 5a). Patients under treatment with HMA + V (P_HMA+V preSCT_, rel_postSCT_) showed no significant differences in frequencies of blasts. In both, CR_preSCT_ and CR_postSCT_ less than 3% blasts were found.

Frequences of DC/cells within several stages of patients before vs. after SCT were comparable (Figure 5a). However, frequencies of DC/cells were borderline significantly higher in patients in CR before vs. after SCT (%DC/cells: CR_preSCT_ 6.1 ± 1.2 vs. CR_postSCT_ 4.0 ± 1.7, *p* = 0.0593). Within DCleu/cells, we found higher frequencies in patients with rel_postSCT_ compared to dgn and rel_HMA+V postSCT_ compared to P_HMA+V preSCT_ (Figure 5a).

#### 2.5.2. Composition of (Leukemia-Specific) Adaptive Cells of the Immune System

Frequencies of T/cells (Figure 5b), Tnn/cells or Tβ7/cells (Figure 5b) across all treatment groups were comparable without significant differences but with slightly higher frequencies of immune cells in CR_preSCT_ vs. CR_postSCT_. However, we found significantly higher frequencies of Tcm/T (Figure 5b) in CR_preSCT_ compared to CR_postSCT_ (%Tcm/T: CR_preSCT_ 24.3 ± 9.1 vs. CR_postSCT_ 12.3 ± 10.7, *p* = 0.0380). Further, we found (highly) significantly lower frequencies of T_IFNg_/cells (Figure 5b) in all treatment groups before vs. after SCT (e.g., %T_IFNg_/cells: dgn 0.6 ± 0.3 vs. rel_postSCT_ 3.9 ± 1.6, *p* = 0.0002; CR_preSCT_ 2.0 ± 0.6 vs. CR_postSCT_ 4.0 ± 2.3, *p* = 0.0343). In contrast, frequencies of antitumor Tβ7_IFNg_/T (Figure 5b) were (highly) significantly higher in patients before vs. after SCT (e.g., %Tβ7_IFNg_/Tβ7: dgn 41.8 ± 27.0 vs. rel 10.1 ± 4.5, *p* = 0.0038; CR_preSCT_ 52.9 ± 22.2 vs. CR_postSCT_ 25.9 ± 17.9, *p* = 0.0192).

We found (borderline) significantly higher frequencies of CIK/cells and iNKT/cells (Figure 5c) in patients at dgn compared to rel_postSCT_ as well as (highly) significantly higher frequencies of CIK/cells, iNKT/cells and iNKT_107a_/cells (Figure 5c) in patients with CR_preSCT_ compared to patients with CR_postSCT_ (e.g., %iNKT/cells: CR_preSCT_ 13.1 ± 12.0 vs. CR_postSCT_ 2.6 ± 1.7, *p* = 0.0046).

## 3. Discussion

### 3.1. Prognosis and Therapeutic Options in Patients with AML

Up to 80% of patients with AML achieve a CR after induction therapy, however 30–80% of them relapse in the following two years [4,43]. Older and unfit patients not eligible for intensive chemotherapy in part benefit from HMA + V, achieving a CR rate of 66.4% with a median overall survival (OS) of 14.7 months [44]. Patients with mutations within the FMS-like tyrosine kinase 3 gene (FLT3), treated with midostaurin achieve a CR in 58.9% of cases and a median OS of 74.7 months [6]. Antibody-based treatments complement the available treatment options. Up to 73% of patients treated with GO achieve CR with a median OS of 34 months. However, relapse occurs in 35% of patients [9]. In summary, there are various factors that influence the individual prognosis of patients at first diagnosis, either positively (e.g., age < 55 years, Karnofsky index > 60%) or negatively (e.g., female gender, high leukocyte count, low hemoglobin concentration, CD56^+^ expression on blasts) [45].

### 3.2. Rationale Behind the Grouping of Study Cohorts and Individual Analytical Variations

In a retrospective analysis of 17 patients’ samples, we analyzed the composition of immune cells of AML patients before SCT in various stages of the disease and in the course of the disease and treatment. Data were categorized according to their stage of the disease stage and the applied treatment regimen. Patients were grouped according to stages and treatment groups: at diagnosis without treatment (dgn), in persistent disease under treatment with chemotherapeutic agents (P_Chemo_), hypomethylating agents with or without venetoclax (P_HMA+V_) or KitM (P_KitM_) and in complete remission (CR). Finally, data obtained before SCT was compared with data after SCT.

Although the statistical power of our retrospective study including 17 patients under non-standardized conditions is limited, our results point to some prognostically relevant findings: some cell types are associated with describe differences in stages of the disease describe or allow differentiations of different ‘modes of action’ of therapies, what could be predictive for prognosis. These results have to be confirmed in (randomized) clinical trials, that include more patients in different stages of the disease, in standardized treatment groups and at standardized time points of analyses.

We describe effects and can deduce the ‘mode of action’ of the new drug ‘Kit M’ in some patients included in our cohorts: We could demonstrate (leukemia specific) activation of effector and memory immune cells, that could contribute to eliminate (residual) blasts and stabilize the disease or remissions. Moreover, hematological benefits for these patients could be demonstrated [13,14].

### 3.3. Effects of Different Treatment Options on the Induction of a (Leukemia-Specific) Immune Response—With a Special Focus on the Prognostical Relevance for Patients

#### 3.3.1. Myeloid Cells

Antigen presenting DCs play an important role in the modulation of the innate and adaptive immune response and consequently, in the fight against tumors or pathogens [46]. DCleu are blast derived DC and express leukemic together with costimulatory antigens, leading to an activation of the immune system with a targeted immune response against the leukemic cells [13,47]. We were able to demonstrate that the frequencies of DC/cells did not differ significantly in our cohort at dgn or under various treatment strategies but were highest in CR (Figure 1a). DC-based immunotherapy has already shown good clinical success in achieving and maintaining CR, so increased endogenous or induced frequencies of DC/cell also suggest a better outcome and prognosis for patients [48]. We found (significantly) lower frequencies of DCleu/cells in P_HMA+V_ compared to dgn and P_Chemo_, and highest frequencies in CR (Figure 1a). Further, we demonstrate that treatment with KitM (GM-CSF and PGE1) induced therapy-associated blast cell conversion to DCleu [13]. Since DCleu are derived from leukemic cells, targeted inhibition of antiapoptotic BCL2 by venetoclax could lead to targeted blast cell death and thus reduce their potential to differentiate into DCleu in P_HMA+V_ [49]. This could lead to a limitation of the effect of KitM in patients pretreated with venetoclax.

The (highly therapy refractory) patient who received individualized treatment with KitM exhibited the highest frequencies of DCleu/cells (despite elevated frequencies of blasts) in this refractory patient (Figure 1a). This confirms the stimulating effect of KitM to induce DCleu, as shown already ex vivo [25] or in vivo in leukemic rats and three refractory end stage patients treated with KitM [13,14] settings before. An induction of blast proliferation under KitM treatment was not observed [13,14].

#### 3.3.2. Adaptive Immune System

Our patient cohorts were characterized by comparable frequencies of T cells—with exception of the KitM treated patient, presenting with higher T cell frequencies (Figure 1b). The highest frequencies of (leukemia-specific) activated and antitumor-relevant T cells were observed during CR or under KitM treatment (Figure 1b,c). This induction, particularly within the T137/T cell subset, suggests KitM induced and DC-mediated T cell proliferation inducing effects [50,51]. CD137^+^ (also known as 4-1BB) functions as a costimulatory (DC-mediated) signal for T cells, promoting their activation and resulting in enhanced anti-tumor responses [40,52]. T137 were found in KitM pretreated (DC inducing) patients thereby confirming that leukemia-specific T cell activity can be augmented by leukemia-derived DC through targeted modulation of the 4-1BB pathway [53].

Frequencies of leukemia-specific T_IFNg_/cells (Figure 1b) and T4^+^_IFNg_/cells (Figure 1c) were highly significantly higher in CR compared to dgn, indicating effective reconstitution of leukemia-specific T-cells in CR, thereby confirming previous data: patients who had received immunotherapy and with higher frequencies of leukemia-specific CD8^+^ T cells maintained a stable CR. These data underscore the significance of leukemia-specific T cells to stabilize remissions and highlights the critical role of their monitoring in immunotherapeutic strategies [54]. Highest frequencies of leukemia-specific T-cells (T_107a_/T, Figure 1b) were detected within the patient treated with KitM or in CR. Both Tβ7/T (Figure 1c) and Tgd/T (Figure 1c) were reconstituted during treatment, reaching their highest frequencies in CR. These findings confirm the important role of (KitM or other) treatments to induce tumor-targeted (in our patients cohort—leukemia-specific) immune responses [31,55]: While low frequencies of Tβ7_107a_/cells and Tgd_TNFa_/cells (Figure 1c) were found in dgn and PD, the frequencies in CR were significantly higher compared to dgn. This observation suggests a potential stimulatory effect of (KitM induced) DCleu to promote leukemia specific T-cell regeneration thereby confirming previous data [13,14].

Furthermore, the results suggest the provision of a (leukemia-specific) immunological memory especially in patients treated with KitM and in CR (Figure 1c). This indicates the induction of immunological memory as part of a DC-mediated function [56]. Significantly higher frequencies of (leukemia-specific) effector (Tem_107a_/Tem, Figure 1c) and central (Tcm/T, Figure 1c) memory T cells were observed in CR compared to dgn and in KitM treated patients.

Increased Treg frequencies are found at first dgn of AML (compared to healthy individuals) and are associated with poor prognosis. Depletion of Tregs have been shown to restore antitumor immunity [57,58,59]. Here we show that patients treated with costimulatory HMA + V or KitM or patients in CR presented the lowest frequencies of (leukemia-specific) Treg (Figure 1d). CTLA4 (CD152^+^) is a well-known inhibitory checkpoint antigen, responsible for immune regulation [60]. Frequences of T152/cells and of Treg in our cohort were comparable (Figure 1d) and lower in all patients in PD and in CR compared to patients at dgn. It has already been demonstrated that a high expression of CTLA4 together with lymphocyte activation gene 3 (LAG3) on T cells at first dgn could be considered a prognostically unfavorable factor for disease free survival [61]. This may indicate an enabled and enhanced antileukemic immune response under therapies reducing checkpoint marker expressing cells and in CR—as a consequence of low Treg and T152 counts.

#### 3.3.3. Innate Immune System

Previous studies have shown that frequencies of NK/cells are reduced in patients with AML compared to healthy probands. Further, NK cell frequencies lower than 9.5% at diagnosis were shown to be associated with an increased risk of relapse [62,63]. Older patients in a pilot clinical trial also benefited from treatment with autologous cytokine-induced killer (CIK) cells combined with recombinant human interleukin-2 (rhIL-2) [64]. In our analysis, CIK/cells, NK/cells (Figure 1e) and iNKT/cells (Figure 1e) were low in all groups compared—except in the KitM treated patients. Leukemia-specific NK_IFNg_/cells (Figure 1e), CIK_IFNg_/cells (Figure 1e) or iNKT_107a_/cells (Figure 1e) cells were most increased in KitM treated patients as well as in patients in CR. DCs have activating influences on the adaptive as well as the innate immune system (especially NK cells) via various cytokines (e.g., IL-12) [65]. Activation of the innate immune system can overcome immune tolerance and promote clinically meaningful antileukemic immunity, particularly through the activity of leukemia-specific innate immune cells [66,67]. Our data point to a KitM induced DC/DCleu-mediated activation of (leukemia-specific) innate cells as shown before in ex vivo settings or in vivo after treatment of leukemically diseased rats or refractory patients [13,14,25,37].

### 3.4. Significance of Individual (Leukemia-Specific) Immune Cells in the Course of the Disease and Treatment

We exemplary present data obtained in two patients in the course of their disease and treatment.

#### 3.4.1. Myeloid Cells

Ex vivo and in vivo studies have already demonstrated that DCleu induce antileukemic effector and memory T cell effects [14,32,47,50]. Refractory Patient 1601 (Figure 2a) received immunomodulatory KitM treatment at relapse. Following an initial increase in DCleu, a rapid and progressive decline in DCleu was observed approximately seven days after treatment initiation under cortisone treatment (applied for a pre-existing COPD), shortly before the patient’s death [13]. In contrast, patient 1618 (Figure 2a), who presented with comparable DCleu frequencies at diagnosis to that seen in patient 1601 at relapse, achieved CR and a 2.5-fold increase in DCleu under chemotherapeutic treatment, with this elevated level maintained for over 100 days.

#### 3.4.2. Immune Cells

Higher frequencies of leukemia-specific immune cells are associated with a favorable outcome in AML patients [54,68]. While patient 1618 exhibited a progressive increase in leukemia-specific adaptive immune cells (e.g., Tβ7_IFNg_/cells) in CR (Figure 2b), patient 1601 demonstrated a gradual decrease in frequencies of Tβ7_IFNg_/cells following an initial increase under KitM treatment (Figure 2b). Leukemia-specific innate immune cells displayed a comparable response pattern to the adaptive immune cells, albeit with a temporal delay. In 1601 (Figure 2b) an increase in (leukemia-specific) NK/cells and iNKT_107a_/cells was observed by day 14 of treatment with KitM, followed by a subsequent decline of these cells followed by the patient’s death. In 1618 (Figure 2b), a slow but sustained increase in (leukemia-specific) NK/cells and iNKT_107a_/cells occurred only after approximately 100 days of treatment. It has been demonstrated that AML patients display reduced numbers of NK cells in peripheral blood, together with an immature NK cell profile. Both factors are associated with adverse prognosis and reduced OS [69].

These observations support the impact of DCleu and (leukemia-specific) immune cells on patients’ outcomes. Although we present interesting observations in the course of the disease, there is a lack of systematic analyses of blood cell compositions at defined timepoints and in defined subgroups (sorted by age, ELN risk, etc.). However, non-immunogenic factors also influence clinical outcomes (e.g., age, mutations, previous illnesses, long-term medication) but were not further addressed in this analysis.

### 3.5. (Leukemia-Specific) Immune and Myeloid Cells in Correlation of Response to Induction (RTI) Therapy and in Patients Studied at First Dgn with Respect to Patients’ Median Survival

We analyzed the composition of (leukemia-specific) immune cells and myeloid cells (at patients’ first dgn) in relation to their response to induction treatment and with respect to patients’ median survival (3.8 months in our patients’ cohort) over the whole treatment.

Various studies have been conducted with promising results to refine prognostic assessment in patients with AML. Some investigations have focused on the prognostic significance of (epi)genetic alterations within the leukemic microenvironment, deducing 12 prognostically relevant genes [70], while others contribute to risk classification using epigenomic sequencing of CpG methylation patterns [71]. Another approach has focused on the prognostic impact of different immune checkpoints and their regulatory genes, demonstrating that high BATF and low EGR1 expression in mononuclear cells were associated with reduced survival [72].

Whereas these strategies use (epi)genetic profiling as prognostic markers our approach focused on immunological marker profiling, given that the immune system undergoes dynamic changes in response to disease and therapy. Future studies have to deduce the roles of these different approaches -e.g., in classifying patients at first diagnosis in (genetically defined) risk groups and e.g., patients in the course of the disease and treated with different (immune modulatory) treatments giving rise to leukemia specific cells according to their chance to respond to different approaches. Moreover, immune cell analyses monitoring might qualify for regular monitoring in defined intervals in the course of the disease. In consequence genetic and or immunological prognostic evaluation or monitoring strategies could contribute to improve survival of AML patients.

#### 3.5.1. Myeloid Cells

We found lower frequencies of blasts (Figure 3a) and comparable frequencies of DCleu/cells (Figure 3a) and DCleu/Bla (Figure 3a) in patients who had responded to induction therapy (responders). Further, patients with lower frequencies of Bla/cells (Figure 4a), higher frequencies of DCleu/DC (Figure 4a) as well as DCleu/Bla (Figure 4a) were characterized by a longer median survival of more than 3.8 months.

We confirm previous studies that high frequencies of blasts at dgn are associated with adverse outcomes including an increased risk of relapse, a reduced OS and CR rates [73,74]: patients with lower frequencies of blasts were characterized by an improved RTI (Figure 3a) and a prolonged median survival compared to those with higher blast frequencies (Figure 4a).

Overall, frequencies of DC/cells, DCleu/cells (Figure 3a) and DCleu/Bla (Figure 3a) were comparable in the responders (RTI) and non responders (without RTI) groups to induction therapy of our cohort. These data indicates that the mentioned therapies (with exception of KitM as DC/DCleu inducing strategy applied to two patients) give rise to comparable frequencies of DC and DCleu. However, we demonstrated that patients with higher levels of DCleu/cells, DCleu/DC (Figure 4a), or DCleu/Bla (Figure 4a) were characterized by a longer survival—thereby confirming previous data, that ex vivo generated DC and DCleu in AML patients relapsed after SCT correlated positively with patients’ response to relapse treatment [75]. Immunomodulatory therapies (e.g., KitM) or DC-based vaccinations have so far demonstrated promising therapeutic potential with a low risk of adverse events [13,14,76,77,78]. This underscores the essential role of dendritic cell-mediated immune defense. Table 3 summarizes the deducted and postulated prognostic relevance of our data.

#### 3.5.2. Immune Cells

We found (borderline significant) higher frequencies of (leukemia-specific) T/cells (Figure 3b), (leukemia-specific) Tcm/cells (Figure 3b) and (leukemia-specific) antitumor-directed Tgd/cells (Figure 3b) and Tβ7_107a_/cells (Figure 3b) as well as leukemia-specific NK_IFNg_/NK (Figure 3c) in patients who had responded to induction therapy. Further, patients in our cohort with higher frequencies of (leukemia-specific) non-naive T cells (Figure 4b) and anti-tumor directed Tβ7/cells (Figure 4b) were characterized by a longer median survival of 3.8 months.

Following chemotherapy, frequencies of lymphocytes in AML patients are reduced and frequencies of T/cells during and within 28d after induction chemotherapy were shown to correlate significantly with improved overall and leukemia-free survival [79]. Consistent with these findings, our data show elevated (leukemia-specific) frequencies of T/cells in patients responding to induction therapy (Figure 3b). We detected higher frequencies of (leukemia-specific) antitumor-directed T cell subtypes (e.g., Tgd) in patients with RTI and a corresponding longer median survival in these patients. This could be attributed to the induced neoplastic regression, going along with the subsequent suppression of tumor cell proliferation [80]. Moreover, patients who survived longer than 3.8 months exhibited a higher number of Tnn/cells. Within this subgroup, we detected significantly increased frequencies of leukemia-specific Tnn_IFNg_/Tnn cells (Figure 4b). In summary, patients in our cohort exhibiting higher frequencies of (leukemia-specific) effector cells had a higher probability to achieve a RTI and demonstrated improved median survival times.

In patients with RTI higher frequencies of early memory CD8^+^ cells have been shown to correlate with improved OS [81]. The memory function of non-naive T lymphocyte subtypes (such as Tem and Tcm) ensures an adequate immune response upon repeated target cell contact and plays an important role in relapse prevention [82]. We confirm that patients with higher frequencies of Tcm/cells (Figure 3b) and leukemia-specific Tcm_107a_/cells (Figure 3b) responded better to induction treatment compared to patients with lower frequencies of (leukemia-specific) memory T cells. These findings suggest that the previously reported prognostic significance of CD8^+^ T cell can be extended to leukemia-specific CD3^+^ T cell populations. Our data showed also a survival benefit for patients with higher frequencies of Tcm/cells (Figure 4b).

Induced (mature) DC and (memory like) NK cells exert antileukemic effects [83] and impaired NK cell function and NK cell frequencies lower than 9.5% at diagnosis have been shown to correlate with an increased risk of relapse [63,84]. In our patient cohort frequencies of ‘overall’ frequencies of NK/cells (Figure 3c) were not predictive, however their composition with respect to leukemia-specific NK cells (NK_IFNg_/NK, Figure 3c) showed a positive correlation with RTI. This highlights their potential as both prognostic markers and effector cells to target AML [85]. Not only frequencies of NK/cells, but also their functionality and exhaustion status are known to correlate with patients’ outcomes [86,87]. Further, we contribute additional data highlighting the role of DC/DCleu to activate (leukemia-specific) T and NK cell subsets, giving rise to antileukemic effects as well as memory cells to fight reoccurring leukemic cells. Therefore we might deduce prognostically relevant conclusions, e.g., predictions of relapses or patients’ responses to induction therapy and survival. Table 3 summarizes the deduced and postulated prognostic relevance of our data.

### 3.6. Effects of Different Treatment Options on the (Leukemia-Specific) Immune Response in Patients Before vs. After SCT

Allogeneic stem cell transplantation (SCT) remains an important and effective treatment option for patients with AML. However, even after SCT, approximately 40% of patients suffer relapses [88,89]. Anand et al., 2025 [88] correlated frequencies of (leukemia-specific) immune cells after SCT under the influence of different treatment strategies in relapsed patients after SCT with patients’ disease progression. Here we compared compositions of immune cells in various stages and treatment groups of the disease before (Dgn, P_HMA+V preSCT_, CR_preSCT_) with findings after SCT (Rel_postSCT_, Rel_HMA+V postSCT_, CR_postSCT_).

#### 3.6.1. Myeloid Cells

Patients with leukemia should be referred to a multidisciplinary treatment center with systematic lifelong follow-up [90], which enables the early detection and treatment of blast proliferation. Rel_postSCT_ patients (most of them included in early stages of relapse) were characterized by lower frequencies of blasts compared to patients at dgn.

Frequencies in both DC/cells (Figure 5a) and DCleu/cells (Figure 5a) were slightly higher in patients with rel_postSCT_ and rel_HMA+V postSCT_. These subgroups may point to a modest immunological advantage, potentially induced by antileukemic, donor derived and DC-mediated effector cells compared to patients without SCT [13,14,48]. Higher frequencies of DC were found in patients before vs. after SCT, probably due to higher (KitM induced) frequencies of DC in patient 1482.

#### 3.6.2. Immune Cells

Higher frequencies of leukemia-specific T cells after SCT have been shown to correlate with a better outcome and survival [68,91,92]. In our cohort, the overall immune cell composition in AML patients before vs. after SCT did not differ significantly (Figure 5b). However, frequencies of leukemia-specific T_IFNg_/cells (Figure 5b) were observed to be borderline or even highly significantly higher in patients after SCT (rel_postSCT_, P_HMA+V postSCT_ and CR_postSCT_). These findings might point to a graft vs. leukemia (GvL) related/mediated leukemia-specific adaptive immune response in various stages after SCT [24]. GvL related cells are known to be involved in the elimination of persistent/residual leukemic cells in the recipient by donor derived T cells that remain in the bone marrow [93,94].

Patients post SCT exhibited a homogeneous distribution of frequencies of Tβ7/cells (Figure 5b), but (highly) significantly lower frequencies of Tβ7_IFNg_/Tβ7 (Figure 5b) relative to pre SCT patients. In addition to its antitumor-relevant function, integrin β7 expressing leukocytes are known to mediate their homing to gut-associated lymphoid tissue, thereby contributing to the pathogenesis of acute graft-versus-host disease (GvHD) [95,96]. Alternatively, the observed lower frequencies in leukemia-specific Tβ7_IFNg_/Tβ7 subset post vs. pre SCT may indicate a lower antitumor activity, mediated by Tβ7_IFNg_ cells in this cohort.

The presence of memory T cells appears to positively influence the maintenance of CR, as patients who experienced relapses after SCT exhibited lower levels of Tcm/T (Figure 5b) cells compared to those in CR_preSCT_ and CR_postSCT_ (Figure 5b). These lower Tcm frequencies in patients in CR post vs. pre SCT might be explained by the higher sensitivity of Tcm to T cell depleting treatment (like anti-thymocyte globulin, ATG) in patients treated by SCT to reduce the probability of GvHD [97].

iNKT cells exert antitumor activity against different types of tumors and frequencies of iNKT are significantly reduced in patients with myelodysplastic syndromes (MDS) [16,98]. We observed low frequencies of both iNKT/cells (Figure 5c) and leukemia-specific iNKT_107a_/cells (Figure 5c) in patients in acute phases of the disease before or after SCT. However, higher frequencies of (leukemia-specific) iNKT cells were found in patients in CR before vs. after SCT, confirming their role to stabilize CR as shown before [99]. Moreover, iNKT cell reconstitution typically reaches normal levels approximately one month after SCT and higher frequencies of iNKT cells post SCT are associated with prolonged OS and protection against GvHD as well as relapse [97]. As most of our samples were analyzed within a short period after SCT, a potential iNKT reconstitution probably was not detectable in our cohorts.

## 4. Materials and Methods

### 4.1. Sample Acquisition

Heparinized blood samples were taken with written consent of the patients and in accordance with the approval of the LMU’s ethics committee (No. 19-034). The University Hospital Augsburg, the Diakonie Hospital Stuttgart and the Red Cross Hospital Munich were involved in this study.

### 4.2. Patients’ Characteristics

In this retrospective study, patients with AML before stem cell transplantation (SCT; *n* = 17) were included at initial diagnosis (dgn; *n* = 14) or with persistent disease (PD) after treatment (*n* = 3). All patients initially presented with an average frequency of 39.9 ± 19.8% (patients at dgn 36.8 ± 19.6%; patients in PD 54.3 ± 13.4%) blasts. The mean age of all patients was 62.5 ± 14.3 years (patients at dgn 59.8 ± 14.4 years; patients in PD 75.5 ± 0.5 years). The male to female ratio of all patients was 1:1.25. Patients were classified by etiology (pAML: *n* = 10; sAML: *n* = 7), ELN risk groups (favorable risk: *n* = 4; intermediate risk: *n* = 4; adverse risk: *n* = 6) in patients at first dgn and responders and no responders to induction therapy (RTI: *n* = 7; no RTI: *n* = 7).

Patients’ and immune profiling data obtained after SCT from Anand et al., 2025 [88] were used to compare the immune cell compositions in patients before and after SCT. In this study, patients with AML after SCT (*n* = 14) were included at relapse (rel; *n* = 12), in partial remission (PR; *n* = 1) or in complete remission (CR; *n* = 1). The mean age of the patients was 59.1 ± 11.8 years with a male to female ratio of 1:1.8.

Patients’ characteristics are provided in Table 4 for patients before SCT and for patients after SCT. Information on detailed individual treatments is provided in the supplement (patients before SCT in Supplementary Table S1 and patients after SCT in Supplementary Table S2).

Patients before SCT were grouped in different treatment groups as given in Table 2: patients at dgn, with disease persistence under chemotherapy (P_Chemo_), with persistence under costimulatory treatment [hypomethylating agents + venetoclax (HMA + V; P_HMA+V_) or GM-CSF + Prostaglandin E1 (KitM, P_KitM_)] and in complete remission (CR). Patients in P_Chemo_ had been treated with cytarabine and daunorubicin, but also one patient each received a single dose of ‘sequential high-dose cytosine arabinoside and mitoxantrone’ (S-HAM), hydroxyurea or midostaurin. Treatment with HMA (azacitidine or decitabine) was combined with venetoclax in 4 of 5 patients, one patient only received HMA. One patient treated with KitM also received hydroxyurea at one timepoint. Analyses of blood samples were performed at different and non-standardized times during/shortly after treatment, or between two treatment cycles. Some patients in CR received consolidation treatment at irregular intervals. However, sample collection of our patients was performed in phases without treatment. Exceptions were one patient, who received long-term treatment with ivosidenib as part of a clinical study, and one other patient, who received long-term treatment with KitM as off label use.

Patients after SCT were grouped in different treatment groups as given in Table 2: rel after SCT (rel_postSCT_), rel under HMA and venetoclax after SCT (rel_HMA+V postSCT_) and CR after SCT (CR_postSCT_). Treatment groups were defined as the mode of action of treatment. In case of multiple flowcytometric measurements in one stage group mean values were used for statistical evaluation.

This study focuses in particular on the antileukemic processes during various treatment methods before and after SCT. A comparison with a healthy control group was not performed at this point, since induced or reduced hemopoetic/immune cells after chemo-, drug-, and immune therapy are not relevant in healthy donors. Moreover, leukemia specific cells are not detectable in healthy donors.

### 4.3. Sample Preparation and Quantification of Myeloid/Immune Cells Using Flowcytometric Analysis

Mononuclear cells (MNC) were isolated from heparinized whole blood (WB) using BioColl^®^ density gradient centrifugation (Bio&Sell, Feucht/Nuernberg, Germany). The quantification of leukemia-specific cells was performed on uncultured WB using the following functional assays: degranulation assay (DEG), the intracellular cytokine assay (INCYT) and the cytokine secretion assay (CSA) after stimulation with leukemia-associated antigens (LAA; WT1 and PRAME). The assays offer the advantage of characterizing functional properties on a single-cell level, thereby enabling the characterization of innate and adaptive immune cell subsets in antileukemic responses. Since it has already been shown that INCYT and CSA achieve comparable results, we grouped data obtained with CSA (*n* = 1) with the INCYT group (*n* = 16) [25,30]. Characterization and quantification of (leukemia-specific) immune cells and myeloid cells were performed using fluorochrome-conjugated antibodies and the “Fluorescence Activating Cell Sorting Flow Cytometer” (FACSCalibur TM, Becton Dickinson, New Jersey, USA). For a detailed description of the experimental procedures, see Klauer et al., 2021 [34], Schutti et al., 2024 [25] and the Supplementary Information.

### 4.4. Evaluation of Data and Statistical Methods

The data obtained by flowcytometry were analyzed with BD Cell Quest™ Pro Software version 6.1. Graphics were performed with the “GraphPad Prism 10” software. The data analyzed were shown as median or mean ± standard deviation (SD). Statistical analyses using the nonparametric Kruskal-Wallis test (P_KW_) and the nonparametric Mann-Whitney U-test were performed using GraphPad Software “Prism 10” and Microsoft 365 Software “Excel”. For patients with more than one measurement during the course of the disease or treatment, the mean value of these measurements was used for further evaluation. Response to induction (RTI) therapy was finalized after latest 21 days of treatment [73,100]. Further, correlations between frequencies of immune cells and RTI were evaluated. To correlate patients’ survival with frequencies of immune cells, we analyzed the period from the first time until three months after the last cell monitoring of individual patients. The median survival time of patients in our study was 3.8 months between the first cell monitoring and the end of the individual observation period (e.g., due to SCT, death or discontinuation of treatment). Finally, we compared the cell composition in patients before and after SCT to find out whether these results could be prognostically relevant.

Differences with a *p*-value of ≤0.005 were considered as “highly significantly” (***) different, a *p*-value of ≤0.05 as “significantly” (**) different, a *p*-value of ≤0.1 as “borderline significantly” (*) different and a *p*-value of >0.1 as “not significantly” different.

## 5. Conclusions

This retrospective analysis highlights the significance of monitoring the composition of (leukemia-specific) immune and myeloid cells in different stages and treatment groups (before or after SCT) to gain information about (specifically treatment associated) reduced or induced cell subsets and their potential to predict prognosis. The data confirm that higher frequencies of leukemia-derived DC (DCleu) and (leukemia-specific) immune cells, particularly Tcm, Tβ7, Tgd and NK cells are associated with improved clinical outcomes, prolonged relapse-free survival and enhanced responses to induction therapy. Furthermore, immunomodulatory treatment with KitM demonstrates a potent opportunity to induce DCleu and augment both (leukemia-specific) adaptive and innate effector and memory immune responses, underscoring its therapeutic potential to stabilize persisting or refractory disease as well as CR before or after SCT.

Our findings emphasize the significance of immunological profiling in AML patients to guide strategies to detect and quantify relevant (leukemia specific) immune cells in the course of their disease, and to deduce cellular/clinical effects of different therapies, including ‘response modifiers and mediators’ in AML patients. Finally, we contribute to integrate defined immune markers in clinical monitoring and treatment managing strategies to improve the outcomes of patients (e.g., by soon therapeutic interventions). Further studies have to include more patients with defined treatment regimens and schedules as well as additional (activating/suppressive) cell markers to validate their values as biomarkers to predict prognosis or to optimize (immunomodulatory) interventions in AML treatment.

## Figures and Tables

**Table 1:** Cells and cell subsets as evaluated by flow cytometry.

Cell Type	Name of Subgroups	Abbreviation of Subgroups	Surface Marker	Abbreviation	Reference
**Myeloid cells**					
Blast cells	Leukemic blasts	Bla	Bla^+^ (CD15^+^, CD34^+^, CD117^+^)	Bla/cells	[26]
Dendritic cells	Dendritic cells	DC	DC^+^ (CD80^+^, CD206^+^)	DC/cells	[25]
	Leukemia derived DC	DCleu	DC^+^Bla^+^	DCleu/cells	[25]
				DCleu/Bla	[25]
				DCleu/DC	[25]
**Immune reactive cells**					
Innate immune system	NK cells	NK	CD3^−^CD56^+^	NK/cells	[27]
	CIK cells	CIK	CD3^+^CD56^+^	CIK/cells	[27]
	Invariant natural killer T cells	iNKT	6B11^+^	iNKT/cells	[28]
Adaptive immune system	CD3^+^ pan T cells	T	CD3^+^	T/cells	[29]
	CD4^+^coexpressing T cells	T4^+^	CD3^+^CD4^+^	T4^+^/T and cells	[29]
	CD8^+^coexpressing T cells	T4^−^	CD3^+^CD4^−^	T4^−^/T and cells	[29]
	Non-naive T cells	Tnn	CD3^+^CD45RO^+^	Tnn/T and cells	[30]
	Effector memory T cells	Tem	CD3^+^CD45RO^+^CD197^−^	Tem/T and cells	[27]
	Central memory T cells	Tcm	CD3^+^CD45RO^+^CD197^+^	Tcm/T and cells	[30]
	γδ T cells	Tgd	CD3^+^TCRgd^+^	Tgd/T and cells	[31]
	Integrine β7 T cells	Tβ7	CD3^+^Intβ7^+^	Tβ7/T and cells	[31]
	Regulatory T cells	Treg	CD3^+^CD4^+^CD25^++^CD127^(+)^	Treg/T and cells	[32]
	CTLA4 expressing T cells	T152	CD3^+^CD152^+^	T152/T and cells	[33]
	4-1BB expressing T cells	T137	CD3^+^CD137^+^	T137/T and cells	[34]
	CD40L expressing T cells	T154	CD3^+^CD154^+^	T154/T and cells	[35]
**Intracellularly IFNg producing cells**					
Innate immune cells	NK cells	NK_IFNg_	IFNg^+^CD3^−^CD56^+^	NK_IFNg_/NK and cells	[25]
	CIK cells	CIK_IFNg_	IFNg^+^CD3^+^CD56^+^	CIK_IFNg_/CIK and cells	[25]
Adaptive immune system	CD3^+^ pan T cells	T_IFNg_	IFNg^+^CD3^+^	T_IFNg_/T and cells	[25]
	CD4^+^-coexpressing T cells	T4^+^_IFNg_	IFNg^+^CD3^+^CD4^+^	T4^+^_IFNg_/T4^+^ and cells	[25]
	CD8^+^-coexpressing T cells	T4^−^_IFNg_	IFNg^+^CD3^+^CD4^−^	T4^−^_IFNg_/T4^−^ and cells	[25]
	Non-naive T cells	Tnn _IFNg_	IFNg^+^CD3^+^CD45RO^+^	Tnn_IFNg_/Tnn and cells	[25]
	Effector memory T cells	Tem_IFNg_	IFNg^+^CD3^+^CD45RO^+^CD197^−^	Tem_IFNg_/Tem and cells	[25]
	Central memory T cells	Tcm_IFNg_	IFNg^+^CD3^+^CD45RO^+^CD197^+^	Tcm_IFNg_/Tcm and cells	[25]
	γδ T cells	Tgd_TNFα_	TNFα^+^CD3^+^TCRgd^+^	Tgd_TNFα_/T and cells	[36]
	Integrine β7 T cells	Tβ7_IFNg_	IFNg^+^CD3^+^Intβ7^+^	Tβ7_IFNg_/Tβ7 and cells	[31]
**Degranulating cells (CD107a)**					
Innate immune cells	NK cells	NK_107a_	CD107a^+^CD3^−^CD56^+^	NK_107a_/NK and cells	[25]
	CIK cells	CIK_107a_	CD107a^+^CD3^+^CD56^+^	CIK_107a_/CIK and cells	[25]
	Invariant natural killer T cells	iNKT_107a_	CD107a^+^6B11^+^	iNKT_107a_/iNKT and cells	[37]
Adaptive immune system	CD3^+^ pan T cells	T_107a_	CD107a^+^CD3^+^	T_107a_/T and cells	[25]
	Non-naive T cells	Tnn_107a_	CD107a^+^CD3^+^CD45RO^+^	Tnn_107a_/Tnn and cells	[25]
	Effector memory T cells	Tem_107a_	CD107a^+^CD3^+^CD45RO^+^CD197^−^	Tem_107a_/Tem and cells	[25]
	Central memory T cells	Tcm_107a_	CD107a^+^CD3^+^CD45RO^+^CD197^+^	Tcm_107a_/Tcm and cells	[25]
	γδ T cells	Tgd_107a_	CD107a^+^CD3^+^TCRgd^+^	TCRγδ_107a_/TCRγδ and cells	[32,36]
	Integrine β7 T cells	Tβ7_107a_	CD107a^+^CD3^+^Intβ7^+^	Tβ7_107a_/Tβ7 and cells	[31]
	Regulatory T cells	Treg_107a_	CD107a^+^CD3^+^CD4^+^CD25^++^CD127^(+)^	Treg_107a_/Treg and cells	[32]

The reference to “cells” relates to MNC in the lymphocyte gate.

**Table 3:** Synopsis of the most important potentially relevant findings deduced from our patient cohorts.

Cell Types:	Finding and Mode of Action:	Associated with favorable Prognosis
**Myeloid cells**	- Blasts displace healthy blood cells - DC induced after blast modulatory/DC inducing treatment (e.g., KitM); connect innate and adaptive immune system; responsible for antigen presentation and induction of specific memory - DCleu induced after blast modulatory treatment; leads to presentation of patient-individual leukemic antigens	- Blasts: lower counts at dgn improves survival and RTI - DC/DCleu: higher counts at dgn improves survival
**(leukemia-specific) adaptive immune effector, memory and regulatory cells**	(LEUKEMIA SPECIFIC) EFFECTOR CELLS - T_IFNg/107a_: leukemia specific T cells mediate antitumor reactions - antitumor-relevant Tgd/Tβ7: induced in part by DC; responsible for inhibition of tumor cell (proliferation) - T137: induced by DC; responsible for DC/DCleu mediated immune responses; induced by KitM (LEUKEMIA SPECIFIC) MEMORY CELLS - Tcm/Tem: result as DC/DCleu effects; responsible for rapid reactivation of anti-infectious/anti leukemic immune cells and relapse prevention (LEUKEMIA SPECIFIC) REGULATORY CELLS - Treg: regulate immune responses, downregulate antileukemic responses; downregulated by KitM - T152: downregulation of T cell activation; downregulated by KitM	- T_IFNg/107a_: higher cell counts at dgn improve RTI, further CR could be maintained - antitumor-relevant cells: higher counts at dgn improves RTI, further CR could be maintained - T137: higher counts could maintain CR - Tcm/Tem: higher counts at dgn improve survival and RTI, further CR could be maintained - Treg/T152: higher counts at dgn and P, indicate active disease with potentially reduced outcome
**(leukemia-specific) innate immune effector cells**	(LEUKEMIA SPECIFIC) EFFECTOR CELLS - NK_IFNg/107a:_ leukemia specific NK cells mediate antitumor reactions, potentially induced by KitM - CIK_IFNg_: leukemia specific effective antitumor response (combining T/NK cell characteristics); potentially induced by KitM - iNKT cells: effective antitumor response (combining T/NK cell characteristics); potentially induced by KitM	- NK_IFNg_: higher cell counts improve RTI, maintain CR, higher counts stabilize CR - CIK_IFNg_: higher counts stabilize CR - iNKT: higher counts stabilize CR

**Table 4:** Patients’ characteristics. Patients before SCT. Patients after SCT (The cohort after SCT is used exclusively for the comparative analysis in Section 3.6).

**Patient** **No.**	**Sex**	**Age**	**Etiology**	**Stage at** **1st Analysis**	**ELN Risk** **Stratification**	**Response** **to Induction**	**Blast Phenotype** **(CD)**	**Blasts in PB (%)**	**Analyzed in Following Treatment Groups**
1599	f	71	pAML	diagnosis	intermediate	No	7, 13, 33, 34, 117	79	Dgn
1603	f	32	pAML	diagnosis	adverse	No	15, 33, 34, 117	47	Dgn, P_Chemo_, P_HMA+V_
1608	f	62	pAML	diagnosis	adverse	Yes	13, 33, 34, 117	55	Dgn, CR
1612	m	77	pAML	diagnosis	adverse	No	13, 34, 117	9	Dgn, P_HMA+V_
1618	m	64	pAML	diagnosis	favorable	Yes	13, 33, 117	29	Dgn, CR
1622	f	49	pAML	diagnosis	favorable	Yes	13, 33, 117	38	Dgn, CR
1627	f	59	pAML	diagnosis	intermediate	No	33, 34, 117	*50*	Dgn, P_Chemo_, P_HMA+V_
1630	m	29	pAML	diagnosis	favorable	No	13, 15, 33, 64, 65, 117	*26*	Dgn, P_Chemo_
1635	m	51	pAML	diagnosis	intermediate	Yes	13, 33, 34, 117	25	Dgn, CR
1609	m	72	sAML	diagnosis	favorable	Yes	7, 13, 33, 117	*22*	Dgn, CR
1624	f	77	sAML	diagnosis	adverse	No	33, 34, 117	38	Dgn, P_Chemo_, P_HMA+V_
1638	m	68	sAML	diagnosis	adverse	No	13, 33, 34, 117	65	Dgn, P_Chemo_
1642	f	64	sAML	diagnosis	adverse	Yes	33, 34, 117	16	Dgn, CR
1651	f	62	sAML	diagnosis	intermediate	Yes	13, 33, 34, 117	16	Dgn
1511	m	76	pAML	persistence			13, 33, 34, 117	42	P_Chemo_
1482	m	75	sAML	persistence			15, 33, 64, 117	48	P_Chemo_, P_KitM_, CR,
1601	f	75	sAML	persistence			4, 7, 33, 117	73	P_KitM_, P_HMA+V_
**Patient** **No.**	**Sex**	**Age**	**WHO Classification**		**No. of SCTs**	**Stage at** **1st Analysis**	**Blast Phenotype** **(CD)**	**Blasts in PB (%)**	**Analyzed in Following Treatment Groups**
1632	f	56	AML with defining genetic abnormalities		2	relapse after SCT	34, 117, 33, 13, 7	7	Rel_postSCT_
1641	f	64	AML, post cytotoxic therapy		2	relapse after SCT	34, 33, 13, 64, 14, 65, 117, 15	5	Rel_postSCT_, P_HMA+V post SCT_, CR_postSCT_
1650	f	64	AML, myelodysplasia-related		2	relapse after SCT	34, 13, 117, 56	1	Rel_postSCT_, P_HMA+V postSCT_, CR_postSCT_
1654	m	71	AML with defining genetic abnormalities		1	relapse after SCT	13, 34, 117	16	Rel_postSCT_, P_HMA+V postSCT_, CR_postSCT_
1655	m	66	AML, myelodysplasia-related		2	relapse after SCT	34, 13, 117,	6	Rel_HMA+V postSCT_, CR_postSCT_
1656	f	42	AML with defining genetic abnormalities		2	relapse after SCT	34, 13, 33, 117, 56	8	Rel_postSCT_, P_HMA+V postSCT_
1658	f	59	AML defined by differentiation		1	relapse after SCT	33, 64, 14, 56	14	Rel_postSCT_
1660	m	70	MDS defined by morphology		2	relapse after SCT	34, 117, 33, 13, 123	6	Rel_postSCT_, P_HMA+V postSCT_, CR_postSCT_
1663	f	46	AML defined by differentiation		3	relapse after SCT	34, 117, 33, 7, 13, 65, 15	5	Rel_postSCT_, CR_postSCT_
1664	m	65	AML with defining genetic abnormalities		2	relapse after SCT	34, 117, 33, 13, 65	3	Rel_HMA+V postSCT_, P_Kit-M postSCT_
1665	m	68	AML, myelodysplasia-related		1	relapse after SCT	33, 34, 13, 15	1	Rel_postSCT_, CR_postSCT_
1674	f	51	AML, myelodysplasia-related		1	relapse after SCT	34, 13, 33, 117	7	Rel_postSCT_, P_HMA+V postSCT_
1640	f	73	AML with defining genetic abnormalities		2	partial remission after SCT	33, 7, 117, 13, 34	1	CR_postSCT_
1603	f	32	AML with recurrent genetic aberrations		1	complete remission after SCT	15, 33, 34, 117		CR_post SCT_

**Legend:** f: female; m: male; AML: acute myeloid leukaemia; pAML: primary AML; sAML: secondary AML; ELN: European Leukaemia Network; CD: Cluster of differentiation; Dgn: first diagnosis; P_Chemo_: persisting disease under chemotherapy; P_HMA+V_: persisting disease under HMA and venetoclax; P_KitM_: persisting disease under KitM; CR: complete remission. Patients’ samples were analyzed in parallel with flow cytometry, degranulation assay (DEG), intracellular cytokine assay (INCYT) or cytokine secretion assay (CSA) (only in patient 1482). CD: Cluster of differentiation; WHO: World Health Organization; SCT: stem cell transplantation; number of SCTs: total number of SCTs since diagnosis; Rel_postSCT_: relapse without therapy; Rel_HMA+V_: relapse under/after hypomethylating agents and/or venetoclax treatment; P_KitM postSCT_: persisting disease under KitM after SCT; CR_postSCT_: complete remission after SCT. Patients’ samples were analyzed in parallel with flow cytometry, degranulation assay (DEG) or intracellular cytokine assay (INCYT).

## Data Availability

Data are contained within the article.

## Supplementary Materials

The following supporting information can be downloaded at: https://www.mdpi.com/article/10.3390/ijms262110336/s1.
