# Supplementary material for: Monitoring of (Leukemia-Specific) Immune Cells in Stages, Treatment Groups and in the Course of Disease and Therapy Contributes to Qualify Antileukemic Potential and Survival in Patients with AML
Source: Int J Mol Sci. 2025 Oct 23;26(21):10336. doi: 10.3390/ijms262110336 (PMC12609274; doi:10.3390/ijms262110336)
Supplement: Supplementary file 1 [file ijms-26-10336-s001.zip › ijms-3875505-supplementary.pdf]

Supplementary tables

Table S1: Individually received therapies in patients before SCT

Patients	Individually stages and received treatments	
**1482**	stage	d1: P2, d14: CR, d35: R2, d60: death
	treatment	d1-4: Hydroxyurea, d4-7: Cytarabine lowdose, d8-d35: KitM, d47: Cytarabine lowdose
**1511**	stage	d1: P1, d318: death
	treatment	d1-7: Daunorubicine with Cytarabine (7+3), d31-38: Cytarabine, d91-220: Midostaurin, d240: Cytarabine lowdose
**1599**	stage	d1: Dgn, d37: CR
	treatment	only one measurement at dgn
**1601**	stage	d1: P2, d33: death
	treatment	d8-25: KitM, d11-13: Cortisone, d18-28: Hydroxyurea
**1603**	stage	d1: Dgn
	treatment	d1-7: Daunorubicine with Cytarabine (7+3), d29-33: HAM-salvage, d80-88: Azacitidine with Venetoclax, d143: SCT
**1608**	stage	d:1 Dgn, d21: CR
	treatment	d1-7: Daunorubicine with Cytarabine (7+3), d37-43: Cytarabine
**1609**	stage	d1: Dgn, d18: CR
	treatment	d1-7: Daunorubicine with Cytarabine (7+3), d28-35: Daunorubicine with Cytarabine (7+3), d62-67: Cytarabine, d96-101: Cytarabine
**1612**	stage	d1: Dgn,
	treatment	d2-9: Azacitidine, d27-34: Azacitidine, d55-62: Azacitidine, d27-81: Venetoclax, d116-122: Azacitidine, d116-150: Venetoclax
**1618**	stage	d1: Dgn, d56: CR
	treatment	d3-10: Daunorubicine with Cytarabine (7+3) and GO, d22-29: Daunorubicine with Cytarabine (7+3), d56-63: Daunorubicine with Cytarabine (1+4) and GO, d12-119: Daunorubicine with Cytarabine (2+4) and GO
**1622**	stage	d1: Dgn, d25: CR
	treatment	d1-8: Daunorubicine with Cytarabine (7+3), d38-43: Cytarabine, d73-78: Cytarabine, d115-120: Cytarabine

**1624**	stage<br>treatment	d1: Dgn, d327: death<br>d1-7: Azacitidine, d22-28: Azacitidine with Venetoclax, d70-76: Azacitidine with Venetoclax, d120-126: Azacitidine with Venetoclax, d170-176: Azacitidine with Venetoclax, d245-250: Cytarabine, d280-285: Cytarabine
**1627**	stage<br>treatment	d1: Dgn<br>d1-7: Daunorubicine with Cytarabine (7+3) and GO, d83-87: Decitabine, d111-115: Decitabine with Venetoclax, d194: SCT
**1630**	stage<br>treatment	d1: Dgn, d58: CR, d1332: R1, 1337: death<br>d1-7: Daunorubicine with Cytarabine (7+3) and GO
**1635**	stage<br>treatment	d1: Dgn, d26: CR<br>d1-7: Daunorubicine with Cytarabine (7+3), d41-48: Daunorubicine with Cytarabine (7+3), d85-88: Cytarabine, d135-138: Cytarabine, d182-185: Cytarabine, d1-945: Ivosidenib
**1638**	stage<br>treatment	d1: Dgn, d39: death<br>d6-11: Daunorubicine with Cytarabine
**1642**	stage<br>treatment	d1: Dgn, d28: CR<br>d3-10: Daunorubicine with Cytarabine (7+3), d45-51: Cytarabine, d79-85: Cytarabine, d134-137: Cytarabine, d254: SCT
**1651**	stage<br>treatment	d1: Dgn, d19: CR<br>d3-10: Daunorubicine with Cytarabine (7+3), d88: SCT

Legend:

d1: day 1 of immune monitoring; Dgn: diagnosis; P1: persisting disease after dgn; P2: persisting disease after first relapse; R1: first relapse; R2: second relapse; CR: complete remission; HAM: high-dose cytosine arabinoside and mitoxantrone; SCT: allogenic stem cell transplant; GO: gemtuzumab ozogamicin;

Table S2: Individually received therapies in patients after SCT

Patients	Individually received therapies
**1603**	**d0: CR1**<br>d0-59: Cyclosporin A with Mycophenolat, d60-161: Mycophenolatmofetil,
**1632**<br>(only 1st analysis)	**d0: R2**<br>**d52: death**
**1640**	**d0: PR1**<br>d6-10: Vidaza/Venetoclax, d11-32: Venetoclax, d36-40: Vidaza/Venetoclax, d41-56: Venetoclax, d71-75: Vidaza/Venetoclax, d76-91: Venetoclax, d113-120: conditioning followed by SCT,<br>**d147: CR2**<br>d303, d345: DLI,<br>**d364: R2**<br>d370-374: Vidaza/Venetoclax, d375-383: Venetoclax,<br>**d407: PR2**<br>d418-427: Vidaza/Venetoclax, d428-440: Venetoclax, d441-445: Vidaza, d446-447: Vidaza/Venetoclax, d448-461: Venetoclax, d469-473: Vidaza/Venetoclax, d474-483: Venetoclax,<br>**d505: CR3**<br>d513, d528, d541, d589, d603, d617, d631, d653, d675: Nivolumab,<br>**d681: R3**<br>d699-703: Vidaza/Venetoclax, d704-705: Venetoclax, d727-731: Vidaza/Venetoclax, d732-733: Venetoclax, d756-758: Vidaza/Venetoclax, d759-763: Decitabine/Venetoclax, d764-772: Venetoclax, d805-809: Decitabine, d842-846: Decitabine<br>**d869: PR3**<br>**d1272: death**
**1641**	**d0: R1**<br>d1-5: Azacitidine/Venetoclax, d6-12: Venetoclax, d40-46: Azacitidine/Venetoclax, d47-49: Venetoclax, d104-120: conditioning followed by SCT<br>**d148: CR2**
**1650**	**d0: R1**

	d1-5: Vidaza/Venetoclax, d6-12: Venetoclax, d33-37: Vidaza/Venetoclax, d38-46: Venetoclax, d95-99: Vidaza/Venetoclax, d100-108: Venetoclax, d158-162: Vidaza/Venetoclax, d163-171: Venetoclax, d186-190: Vidaza/Venetoclax, d191-199: Venetoclax, d238-246: conditioning followed by SCT<br>**d274: CR2**<br>d467, d491, d519: DLI
**1654**	**d0: R2**<br>d7-11: Vidaza/Venetoclax, d12-34: Venetoclax, d35-39: Vidaza/Venetoclax, d40-64: Venetoclax, d65-69: Vidaza/Venetoclax, d70-90: Venetoclax, d91-95: Vidaza/Venetoclax, d96-125: Venetoclax, d126-130: Vidaza/Venetoclax, d131-132: Venetoclax, d136, d164: DLI<br>**d199: CR3**
**1655**	**d0: R1**<br>d0-4: Decitabine/Venetoclax, d5-22: Venetoclax, d27-36: Decitabine/Venetoclax, d37-d40: Venetoclax, d50: Cytarabin/Daunorubicin, d51-54: Cytarabin, d57-70: conditioning followed by SCT<br>**d98: CR2**<br>d213, d241, d269: DLI
**1656**	**d0: R1**<br>d0-d1: Venetoclax, d2-11: Decitabine/Venetoclax, d12-14: Venetoclax, d44-47: Cytarabin, d49-55: conditioning followed by SCT<br>**d85: CR2**<br>**d143: R2**<br>**d330: death**
**1658**<br>(only 1st analysis)	**d0: R1**<br>**d10: death**
**1660**	**d0: R1**<br>d7-11: Vidaza/Venetoclax, d12-20: Venetoclax, d40-44: Vidaza/Venetoclax, d45-51: Venetoclax, d67-71: Vidaza/Venetoclax, d72-86: Venetoclax,<br>d96-100: Vidaza/Venetoclax, d101-110: Venetoclax, d130-134: Vidaza/Venetoclax, d135-143: Venetoclax, d229-237: conditioning followed by SCT<br>**d263: CR2**<br>**d286: death**
**1663**	**d0: R3**

	d15-19: Decitabine/Venetoclax, d20-28: Venetoclax, d42-46: Decitabine/Venetoclax, d47-55: Venetoclax, d56-62: conditioning followed by SCT<br>**d90: CR4**
**1664**	**d0: R2**<br>d0-4, d28-32, d64-68, d99-103: Vidaza, d120-124, d127-131, d136-140, d143-147, d172-176, d194-198, d210-214, d224-228: Kit-M<br>**d265: Death**
**1665**	d0: R1<br>d19-25: Vidaza/Venetoclax, d47-53: Vidaza/Venetoclax, d54-74: Venetoclax, d75-81: Vidaza/Venetoclax, d89-94: Venetoclax<br>**d95: CR2**<br>d95-104: Venetoclax, d105-111: Vidaza/Venetoclax, d112-142: Venetoclax, d143-149: Vidaza/Venetoclax, d150-170: Venetoclax, d171-177: Venetoclax, d178-194: Venetoclax, d199-205: Vidaza
**1674**	**d0: R1**<br>d6-10: Decitabine<br>**d69: death**

Legend:

d0: day zero of immune monitoring; R1: first relapse; R2: second relapse; R3: third relapse; PR1: first partial remission; PR2: second partial remission; PR2: third partial remission; CR1: first complete remission; CR2: second complete remission; CR3: third complete remission; CR4: fourth complete remission; SCT: stem cell transplantation;

Supplementary materials and methods

1. Characterization and quantification of immune cells using flowcytometric analysis

Analysis of Bla, DC, DCleu and immunoreactive cells (NK, CIK, iNKT, T, $T4^{+}$, $T4^{-}$, Tnn, Tem, Tcm, Tgd, Tβ7, Treg) as well as checkpoint marker-expressing (T152) and co-stimulatory (T137, T154) T cells was performed using the "Fluorescence Activating Cell Sorting Flow Cytometer" (FACSCalibur TM, Becton Dickinson). Various monoclonal antibodies labelled with fluorescein isothiocyanate (FITC), phycoerythrin (PE), phycoerythrin cyanine 7 (PC7) or allophycocyanin (APC) were used for this purpose. The antibodies were provided by the following companies: BioLegend (Koblenz, Germany), Beckman Coulter (Krefeld, Germany) and Becton Dickinson (Heidelberg, Germany). Furthermore, all measurements were performed using isotope control. A minimum of 30 gated mononuclear cells were required. An overview of the analyzed cells and cell subsets is given in **Table 1**. Frequencies of respective cells or subtypes are given referred to subtype or lymphocyte fractions. Gates were set around blasts, DCs or lymphocytes as described before [25].

2. Leukemia-specific assays (INCYT and DEG)

Intracellularly IFNg or TNFα producing cells were quantified using the INCYT assay. Uncultured WB was stimulated with the LAA "Wilms Tumor 1" (WT1; PepTivator®, Miltenyi Biotec, Bergisch Gladbach, Germany) and "preferentially expressed antigen in melanoma" (PRAME; PepTivator®, Miltenyi Biotec, Bergisch Gladbach, Germany). Spontaneous cytokine secretion was prevented by using Breveldin (BioLegend, Koblenz, Germany, c=5µg/ml), fixation and permeabilization was realized with Medium A and B (FiX&PERM, Thermo Fisher Scientific, Waltham, USA). Subsequently, the blood was incubated for 16 hours (37°C, O_2 21%, CO_2 25%) as shown before [25]. In one patient, the cytokine secretion assay (CSA) was used. Here, IFNg binds to the panleukocytic CD45 marker through IFNg catch reagent (Miltenyi Biotec, Bergisch Gladbach, Germany) and is visualized by PE conjugated IFNg detection antibody (Miltenyi Biotec, Bergisch Gladbach, Germany) as shown before [37].

Degranulating (effector) cells were quantified using the DEG assay. As degranulation marker FITC-conjugated antibody against "lysosomal associated membrane protein1" (LAMP1, CD107a) was used after stimulation of immune cells with LAA. To minimize attenuation of CD107a fluorescence, Monensin (BioLegend, Koblenz, Germany, c=2µg/ml) was used and the immune cells consecutively incubated for 16 hours (37°C, O_2 21%, CO_2 25%) as shown before [25].
